# Decoupled Early Time Series Classification Using Varied-Length Feature Augmentation and Gradient Projection Technique

**DOI:** 10.3390/e24101477

**Published:** 2022-10-17

**Authors:** Huiling Chen, Ye Zhang, Aosheng Tian, Yi Hou, Chao Ma, Shilin Zhou

**Affiliations:** College of Electronic Sciences and Technology, National University of Defense Technology, Changsha 410073, China

**Keywords:** early time series classification, varied-length time series classification, early exiting, random length truncation, gradient projection

## Abstract

Early time series classification (ETSC) is crucial for real-world time-sensitive applications. This task aims to classify time series data with least timestamps at the desired accuracy. Early methods used fixed-length time series to train the deep models, and then quit the classification process by setting specific exiting rules. However, these methods may not adapt to the length variation of flow data in ETSC. Recent advances have proposed end-to-end frameworks, which leveraged the Recurrent Neural Networks to handle the varied-length problems, and the exiting subnets for early quitting. Unfortunately, the conflict between the classification and early exiting objectives is not fully considered. To handle these problems, we decouple the ETSC task into the varied-length TSC task and the early exiting task. First, to enhance the adaptive capacity of classification subnets to the data length variation, a feature augmentation module based on random length truncation is proposed. Then, to handle the conflict between classification and early exiting, the gradients of these two tasks are projected into a unified direction. Experimental results on 12 public datasets demonstrate the promising performance of our proposed method.

## 1. Introduction

Due to the widespread application of sensors, a large amount of sequence data is generated in various real-world applications [1,2,3,4]. For some time-sensitive applications [5,6,7,8,9,10,11], such as disaster prediction, gas leakage detection and fault diagnosis, it is crucial to identify the classes of observable time series as accurately and quickly as possible [12,13]. Therefore, early time series classification (ETSC) has high research value.

In the last few years, deep learning has obtained increasing attention in ETSC tasks. Existing deep learning based methods can be classified into the one-stage and two-stage categories, according to the implementation procedures of different models. Since early stages of sequences cannot provide sufficient classification information, one of the main challenges of ETSC is to balance the conflicting objectives of classification and early exiting [14,15,16]. To tackle this problem, some two-stage methods used the whole sequences with fixed-length to train the classifier, and then set exiting rules for early quitting [17,18,19]. These methods handle the conflict between accuracy and earliness by separately optimizing these two targets. However, they may lack full consideration in the length variation of flow data in ETSC, leading to poor classification performance. To this end, some one-stage methods proposed end-to-end frameworks that leveraged the Recurrent Neural Networks (RNNs) to handle the varied-length sequences, and used the exiting subnet to quit classification [20,21,22]. Unfortunately, due to the joint optimization manner of these methods, the conflict between classification and early exiting is underestimated.

**Figure 1:**
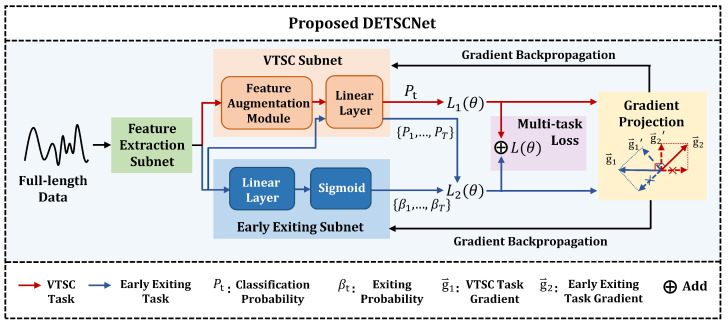
The overall framework of the DETSCNet.

In this paper, we propose an end-to-end framework, named **D**ecoupled **ETSC Net**work (DETSCNet). DETSCNet integrates the advantages of previous one-stage and two-stage methods by decoupling the ETSC task into the variable-length time series classification (VTSC) task and the early exiting task (see Figure 1). To handle the varied-length problem of flow sequences, a feature augmentation module based on random length truncation is designed. This module is embedded into the classification subnet, and truncates complete features at random lengths. Then, the truncated features are passed through the linear layer for classification. Moreover, a multi-task loss function specially designed for the VTSC task is proposed, which further improves the classification performance. Through the designed feature augmentation module and multi-task loss function, the adaptive capabilities of classification subnet to the data length variation can be enhanced.

To balance the conflict between classification and early exiting, a gradient projection technique is proposed. Specifically, we present the gradient distributions of classification task and early exiting task in Section 5.2. As shown, most gradients of these two tasks have opposite directions, which brings difficulties to the optimization of the VTSC and exiting subnets. To handle this problem, we project the conflicting gradients generated by these two subnets into a unified direction. In this way, the conflicting objectives of classification and early exiting can be effectively balanced.

The main contributions of this paper are summarized as follows:

- We propose an end-to-end framework to decouple the ETSC task into VTSC and early exiting, named DETSCNet;
- To enhance the adaptive capabilities of the classification model to the data length variation, a feature augmentation module based on random length truncation and a multi-task loss function specially designed for VTSC are proposed;
- To handle the conflict between the classification and early exiting, a gradient projection technique is designed;
- The proposed method achieves superior performance on 12 public datasets.

## 2. Related Work

In the last few years, many works have performed extensive exploration in ETSC. Among them, the traditional methods leveraged hand-crafted features to train multiple classifiers, and set exiting strategies to quit classification. According to different exiting strategies [15], these methods can be divided into Prefix based [23,24,25,26], Shapelet based [27,28,29,30], and Model based [31,32,33,34,35,36] categories. Although these methods have achieved impressive performance and obtained extensive research, it is difficult to obtain the expert knowledge for constructing hand-crafted features as well as multiple classifiers. Recently, deep learning has achieved promising performance in TSC [37]. In ETSC, it has also raised wide attention [38,39,40]. Compared with the traditional methods, deep learning performs feature extraction and classification schemes automatically. Therefore, this work mainly focuses on the deep learning based methods.

Existing deep learning methods can be classified into two categories, including the two-stage methods and the one-stage methods. By separating the classification task from the early quitting task, The two-stage methods handle the conflicting objectives of classification and early exiting effectively. Min et al. [41] first leveraged the complete time series with fixed length to train the hybrid model composed of the Convolutional Neural Network (CNN) and the Gated Recurrent Unit (GRU), and then set the classification probability threshold for early quitting. However, due to the diversity of data characteristics, accurate thresholds are hard to obtain. To tackle this problem, some methods designed exiting rules to quit the classification process automatically. Sharma et al. [7,17] first used the hybrid model of CNNs and RNNs to extract classification features from complete sequences, and then designed a cost function to learn the suitable exiting threshold automatically. Shekhar et al. [18] calculated the difference between the early classification cost and the misclassification cost. The classifier exits only when the difference is positive. Although existing two-stage methods avoid the conflict between classification and early exiting by optimizing these objectives separately, the classification models of these methods are usually trained with fixed-length sequences, underestimating the varying lengths of flow data.

Recently, some one-stage methods proposed end-to-end frameworks, which leveraged the RNNs to handle the length variation problem, and the exiting subnet for early quitting. Marc et al. [20,21] used the Long Short Term Memory (LSTM) to adapt to the varying lengths of flow data. Then, a loss function based on the misclassification and time costs was designed to optimize the classification and exiting subnets jointly. However, the end-to-end optimization process may lack consideration in the conflicting objectives of classification and early exiting.

In summary, the explorations of deep learning-based ETSC methods are far from sufficient. Specifically, the two-stage methods handle the conflict between the classification and the exiting processes, while underestimating the varying lengths of flow data. On the contrary, the one-stage methods leverage the RNNs to adapt to the length variation of signals, while failing to effectively balance the conflicting objectives of classification and early exiting. This motivates us to integrate the advantages of both one-stage and two-stage methods and avoid the defects of them.

## 3. Methods

### 3.1. Overview

This section introduces our proposed model, named DETSCNet. In Section 3.2, the overall architecture of DETSCNet is introduced. Then, we separately provide our core contributions in Section 3.3 and Section 3.4. Specifically, in Section 3.3, a VTSC subnet based on feature augmentation module is proposed. Through this subnet, DETSCNet better adapts to the data length variation. Moreover, a multi-task loss function specially designed for the VTSC task is illustrated. In Section 3.4, the gradient projection technique used to unify the directions of conflicting gradients between VTSC and early exiting tasks is described.

### 3.2. The Architecture of DETSCNet

**Figure 2:**
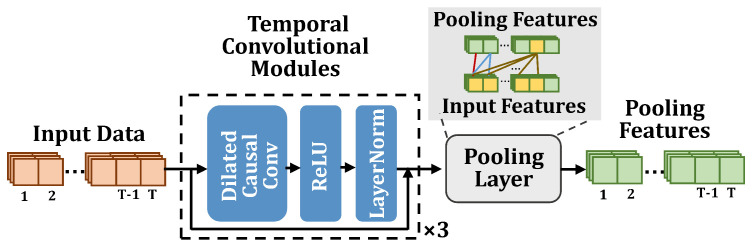
The architecture of the feature extraction subnet.

This section briefly introduces the architecture of our proposed DETSCNet. The overall framework of DETSCNet is presented in Figure 1. As shown, the extracted features are first fed into the feature extraction subnet, whose structure is presented in Figure 2. As shown, this subnet is mainly composed of the temporal convolutional modules [42] and an average pooling layer. A temporal convolutional module further comprises the dilated causal convolution, Relu, and LayerNorm layer. We first use the temporal convolutional modules to extract features from complete sequences. Then, the average pooling operation is performed at each timestamp of the truncated features. In this way, the features of previous timestamps are aggregated to the current timestamp. Next, these pooling features are separately fed into the VTSC subnet and the early exiting subnet, to obtain the classification probability at timestamp *t* ($P_{t}$), the classification probabilities of all timestamps ($P_{1},\ldots ,P_{T}$), and the exiting probabilities of all timestamps ($\beta_{1},\ldots ,\beta_{T}$). Among them, $P_{t}$ is used to calculate the VTSC loss ($L_{1}\left(\theta \right)$, red line in Figure 1). $\left\{P_{1},\ldots ,P_{T}\right\}$ and $\left\{\beta_{1},\ldots ,\beta_{T}\right\}$ are used to calculate early exiting loss ($L_{2}\left(\theta \right)$, blue line in Figure 1). Finally, the multi-task loss function (L(θ)) composed of $L_{1}\left(\theta \right)$ and $L_{2}\left(\theta \right)$ is applied to optimize DETSCNet, using the gradient projection technique to project the conflicting gradients of these two tasks into a unified direction.

### 3.3. Varied-Length Time Series Classification

**Figure 3:**
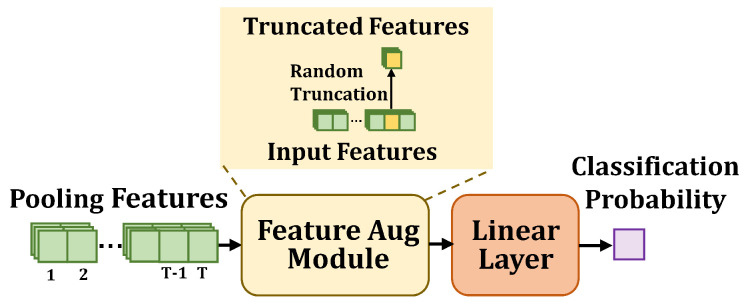
The architecture of the VTSC subnet.

In this subsection, the VTSC subnet with the varied-length feature augmentation module is described. The architecture of the VTSC subnet is presented in Figure 3. Moreover, the multi-task loss function used to optimize the VTSC subnet is also illustrated.

#### 3.3.1. The Varied-Length Feature Augmentation Module

In ETSC, the lengths of the input data increase over time. However, the two-stage methods usually use complete time series with fixed-length to train the models, lacking adaptation to the length variation of flow data.

To handle this problem, a straightforward idea is to perform the feature augmentation by truncating each training sequence to a random length. However, since the samples in a batch have different lengths, the model cannot be trained directly. Therefore, we perform random truncation at the feature level. The architecture of the VTSC subnet based on the feature augmentation module is presented in Figure 3. As shown, the pooling features are first passed through a feature augmentation module. Then, we randomly select the features of a certain timestamp (as the truncated features), and feed them into the linear layer to obtain the classification probability $P_{t}$.

#### 3.3.2. The Multi-Task Loss Function

The multi-task loss function is composed of the VTSC loss and early exiting loss. It is proposed to optimize the VTSC subnet and early exiting subnet in an end-to-end framework. The exiting subnet is a binary classification network used to determine whether to exit or not (see Figure 1). The proposed multi-task loss function L(θ) can be defined as

(1) $$L\left(\theta \right)=L_{1}\left(\theta \right)+L_{2}\left(\theta \right),$$

where θ represents the parameters of our model. $L_{1}\left(\theta \right)$ is a cross-entropy loss function specially added for the VTSC subnet. $L_{2}\left(\theta \right)$ is the loss function designed for the early exiting task. $L_{1}\left(\theta \right)$ and $L_{2}\left(\theta \right)$ can be separately defined as

(2) $$L_{1}\left(\theta \right)=-\frac{1}{N}\sum_{i=1}^{N}\log\left(P^{i}\right),$$

(3) $$L_{2}\left(\theta \right)=\frac{1}{N}\sum_{i=1}^{N}\sum_{t=1}^{T}C_{t}^{i}*\delta_{t}^{i}.$$

$P^{i}$ refers to the probability that the sample *i* is correctly classified at the truncated timestamp, which is obtained by feeding the extracted features to the feature augmentation module and linear layer. *N* is the number of samples in the training set. In Equation (3), $\delta_{t}^{i}$ (conditional probability) and $C_{t}^{i}$ (the exiting condition) are separately defined as

(4) $$\delta_{t}^{i}=\beta_{t}^{i}\prod_{j=1}^{t-1}\left(1-\beta_{j}^{i}\right),$$

(5) $$C_{t}^{i}=a*\left(-\log\left(P_{t}^{i}\right)\right)-\left(1-a\right)*P_{t}^{i}*\left(1-t/T\right).$$

In Equation (4), $\beta_{j}^{i}$ is the exiting probability of the sample *i* at timestamp *j*, which is obtained by feeding the extracted features to the linear layer of the early exiting subnet. $\delta_{t}^{i}$ is the exiting probability at timestamp *t* under the condition that the classifier cannot exit classification before time t. Note that $\delta_{T}^{i}=1-\sum_{t=1}^{T-1}\delta_{t}^{i}$, where *T* refers to the length of the complete time series. In Equation (5), $P_{t}^{i}$ is the probability that the sample *i* is correctly classified at timestamp *t*. It is obtained by feeding the extracted features directly to the linear layers of the VTSC subnet (see the upper blue line in Figure 1). The first term $\left(-\log\left(P_{t}^{i}\right)\right)$ refers to the misclassification penalty, and the second term $P_{t}^{i}*\left(1-t/T\right)$ refers to the reward of correct classification in advance. This exiting condition $C_{t}^{i}$ constructs the association between the exiting subnet and classification subnet via $P_{t}^{i}$. The parameter a∈[0,1] is the balancing parameter used to control the relationship between the early reward and misclassification penalty.

### 3.4. Gradient Projection Technique

In this subsection, the gradient projection technique is introduced in details. To illustrate the conflict between classification and early exiting, the gradient distributions of these two tasks are presented in Figure 8. It can be observed that most gradients of these two tasks have opposite directions. Consequently, it is difficult to optimize the VTSC and exiting subnets effectively.

To handle this problem, we project the conflicting gradients of the two tasks into a unified direction. First, we separately perform backpropagation according to $\nabla L_{1}\left(\theta \right)$ and $\nabla L_{2}\left(\theta \right)$, where θ represents all network parameters. In this way, the gradients of VTSC task ${\vec{g}}_{1}$ and the gradients of early exiting task ${\vec{g}}_{2}$ are obtained. The conflicting gradients are then distinguished by calculating the cosine similarity of ${\vec{g}}_{1}$ and ${\vec{g}}_{2}$ by Equation (6):

(6) $$\cos\left(\varphi \right)=\frac{{\vec{g}}_{1}\cdot {\vec{g}}_{2}}{∥{\vec{g}}_{1}∥\;∥{\vec{g}}_{2}∥}$$

where φ is the angle between the ${\vec{g}}_{1}$ and ${\vec{g}}_{2}$, $∥{\vec{g}}_{1}∥$ indicates the norm of ${\vec{g}}_{1}$. If ${\vec{g}}_{1}$ and ${\vec{g}}_{2}$ are under an unified direction, their cosine similarity is positive. On the contrary, if ${\vec{g}}_{1}$ and ${\vec{g}}_{2}$ are under opposite directions, their cosine similarity is negative. Note that, the gradient projection is performed on those conflicting gradients with negative cosine similarity.

Specifically, ${{\vec{g}}_{1}}^{*}$, ${{\vec{g}}_{2}}^{*}$ can be updated by Equations (7)–(9), and the final projected gradient ${\vec{g}}^{*}$ for backpropagation can be calculated by (10):

(7) $$C=\left\{\begin{array}{cc} 1, & \;\mathrm{if}\;\cos(\varphi )<0\\ 0, & \;\mathrm{if}\;\cos(\varphi )\geq 0 \end{array}\right.,$$

(8) $${{\vec{g}}_{1}}^{*}={\vec{g}}_{1}-C*\frac{{\vec{g}}_{1}\cdot {\vec{g}}_{2}}{{∥{\vec{g}}_{2}∥}^{2}}{\vec{g}}_{2},$$

(9) $${{\vec{g}}_{2}}^{*}={\vec{g}}_{2}-C*\frac{{\vec{g}}_{1}\cdot {\vec{g}}_{2}}{{∥{\vec{g}}_{1}∥}^{2}}{\vec{g}}_{1},$$

(10) $${\vec{g}}^{*}={\vec{g}}_{2}^{*}+{\vec{g}}_{1}^{*}.$$

**Figure 4:**
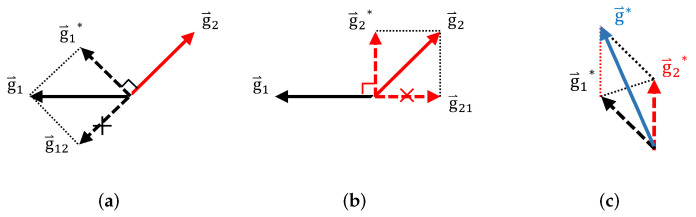
Gradient projection technique of conflicting gradients. (**a**) gradient projection of ${\vec{g}}_{1}$; (**b**) gradient projection of ${\vec{g}}_{2}$; (**c**) obtained projected gradient. In (**a**), ${\vec{g}}_{1}$ is projected to the vertical direction of ${\vec{g}}_{2}$ to obtain ${\vec{g}}_{1}^{*}$. In (**b**), ${\vec{g}}_{2}$ can be obtained in a similar manner. In (**c**), the final projected gradients ${\vec{g}}^{*}$ for backpropagation can be obtained by the sum of ${\vec{g}}_{1}^{*}$ and ${\vec{g}}_{2}^{*}$.

In Equation (8), the second term $\frac{{\vec{g}}_{1}\cdot {\vec{g}}_{2}}{{∥{\vec{g}}_{2}∥}^{2}}{\vec{g}}_{2}$ is the projection of ${\vec{g}}_{1}$ in the opposite direction of ${\vec{g}}_{2}$. We subtract $\frac{{\vec{g}}_{1}\cdot {\vec{g}}_{2}}{{∥{\vec{g}}_{2}∥}^{2}}{\vec{g}}_{2}$ from the original ${\vec{g}}_{1}$ to get the updated ${{\vec{g}}_{1}}^{*}$ (see ${{\vec{g}}_{1}}^{*}$ in Figure 4a). ${\vec{g}}_{2}$ can also be obtained in a similar manner (see ${{\vec{g}}_{2}}^{*}$ in Figure 4b). The final projected gradient ${\vec{g}}^{*}$ for backpropagation can be obtained by the sum of ${\vec{g}}_{1}^{*}$ and ${\vec{g}}_{2}^{*}$. Note that, when *C* in Equation (7) is equal to 0, the non-conflicting gradients remain unchanged (see Equations (8) and (9)). Using the gradient projection technique, the conflict between the VTSC and early exiting subnets in the joint optimization can be handled effectively.

## 4. Experiments

### 4.1. Experimental Setup

#### 4.1.1. Dataset

Twelve commonly used datasets are used for performance evaluation. Ten of them are univariate time series datasets and the remaining two are multivariate time series datasets. These datasets are briefly described as follows.

Univariate time series datasets: We selected 10 commonly used datasets from the UCR datasets [43]. The UCR datasets contain various categories of time series, including smart home, biomedical, etc. These datasets are widely used in ETSC [27,29,44]. These datasets have been z−normalized and the UCR repository provides train and test split sets. The selected datasets and their description are available at http://www.timeseriesclassification.com, accessed on 1 July 2021.

Multivariate time series datasets: The Heterogeneity Human Activity Recognition (HHAR) dataset [45] and Daily and Sports Activities (DSA) dataset [45] are used in our experiments. HHAR and DSA are two widely used human activity recognition datasets [24,25,46]. The early classification of human activities helps to minimize the response time of the system, and improves the user experience [15]. The HHAR dataset was collected from 9 subjects. All the subjects performed 6 activities carrying smartwatches and smartphones from different manufacturers. The readings from the smartphone with device number nexus4_1 were used in our experiments. The original data was segmented by a sliding window of 1200 readings with 25% overlap. The DSA dataset was collected from 8 subjects. Each subject performed 19 activities wearing 5 units of motion sensors on 5 different body parts. We selected 7 activities from these activities and only used 5 motion sensors in our experiments, which is the same as that in Ref. [46]. The data in the HHAR dataset and DSA dataset were randomly divided into training and testing datasets according to a 7:3 ratio.

#### 4.1.2. Training Procedures

All the compared models are implemented in Pytorch 1.9.0 [47] on a computer with Nvidia RTX 3080 GPU. They are optimized using the Adam optimizer. The learning rate was initialized as 1e-3, and the learning rate is halved if the loss of the training dataset does not decrease within 10 epochs. The number of epochs is set to 200. Besides, the balanced parameter *a* in Equation (5) is set to 0.5. The model with the lowest loss on the training dataset is selected for performance evaluation. To establish a fair comparison, the number of network parameters in each comparison model is no less than our proposed model. More detailed configurations of our model are listed in Table 1.

#### 4.1.3. Test Procedures

**Figure 5:**
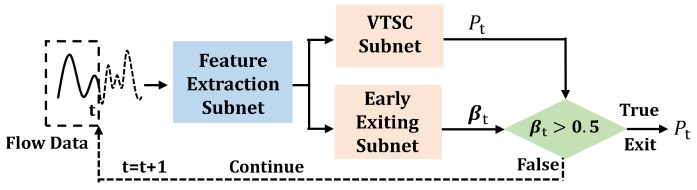
Test procedures. The flow data is first input into feature extraction module. Then the extracted features are separately input into the VTSC subnet and early exiting subnet, to obtain the classification probability $P_{t}$ and exiting probability $\beta_{t}$. If $\beta_{t}$ is greater than 0.5, the classification result at timestamp *t* will be considered as the final predicted category. Otherwise, the sequence data is continuously fed to the classifier over time.

As shown in Figure 5, in the test phase, the flow data is first input into the feature extraction module, and then the extracted features are fed into classification and early exiting subnets. In this way, the classification probability $P_{t}$ and exiting probability $\beta_{t}$ can be obtained separately. Note that the varied-length augmentation feature module was removed in the test phase. If $\beta_{t}$ is greater than 0.5, the classification result at timestamp *t* will be considered as the final predicted category. Otherwise, the sequence data is continuously fed to the classifier over time.

#### 4.1.4. Evaluation Rule

Based on previous work, follow the TEASER [44], the harmonic mean (HM) of earliness and accuracy is used as the evaluation metric in this work. The larger the value of HM, the better the early classification performance of the method. HM is defined as

(11) $$HM=\frac{2\cdot (1-\;\mathrm{earliness}\;)\cdot \;\mathrm{accuracy}\;}{(1-\;\mathrm{earliness}\;)+\;\mathrm{accuracy}\;}.$$

### 4.2. Comparisons of Different Methods

The proposed method (DETSCNet) is compared with four methods, which are listed as follows.

SR2-CF2: The SR2-CF2 model [35] is considered as the baseline method among traditional methods. This method combines a set of probabilistic classifiers together with an exiting rule. For the 10 UCR datasets, we use the same results as those published in their supplementary material, with parameter α = 0.8. For the HHAR and DSA datasets, we cite the results in Ref. [46].

ECLN: An end-to-end framework embedded in the learning decision mechanism is proposed in Ref. [20]. This decision mechanism is implemented by constructing an exiting subnet. The method used LSTM as the backbone network, and proposed a loss function to jointly optimize the parameters of the classification subnet and the exiting subnet.

ETMD: This method combines CNNs and RNNs to develop a hybrid deep learning classifier. Furthermore, a decision strategy is defined to obtain a suitable threshold for exiting [17]. This decision strategy was also used in Ref. [48]. The dynamic decision fusion method fuses the classification results at multiple early moments.

EPTS: To maximize the probability of a correct label as early as possible, this method proposed a loss function and designed a module for gesture recognition to modify the current sequence according to the existing information. Since the module is specially designed for motion information of gesture recognition, we do not use this module when reproducing the code [41].

The quantitative HM scores results of the DETSCNet and four compared methods are provided in Table 2. As shown, our method achieves the best early classification performance on most datasets compared with the competitors. The best performance is highlighted in bold and the second best is highlighted in italics. Some other important observations can also be made from Table 2. First, among the deep learning methods (ECLN, ETMD, EPTS), the early performance of the two-stage methods (ETMD, EPTS) outperforms the one-stage method (ECLN). This may be because the two-stage methods take the conflicts between classification and exiting into account. Second, compared with the traditional method SR2-CF2, EPTS obtains obvious advantages on certain datasets(HHAR, DSA, Twopattens), and achieves comparable performance on other datasets. This may be because the representation ability of handcrafted features is highly dependent on the expert’s experience, limiting the classification performance of SR2-CF2. Besides, our method achieves the best performance on almost all of the datasets, since the data length variation and the conflicts between classification and exiting are effectively handled.

### 4.3. Ablation Study

To demonstrate the effectiveness of the components of the proposed method, the ablation experiments are provided in this section. For fair comparison, the TCNs are used as the backbone network in all ablation experiments. The ablation experiments are divided into two parts. First, the experiments of the VTSC subnet (comprised of feature augmentation module and multi-task loss function) are presented in Section 4.3.1. Then, the experiments of the gradient projection technique are provided in Section 4.3.2.

#### 4.3.1. Ablation Experiments of the VTSC Subnet

To illustrate the effectiveness of the VTSC subnet (comprised of the feature augmentation module and multi-task loss function), several ablation experiments are performed. Specifically, we use the TCN backbone (with loss function in ECLN [20]) as the baseline, and then the baseline with (abbreviated to baseline+FA) and without (baseline) the feature augmentation module and multi-task loss function are compared. The quantitative performance of the baseline and baseline+FA on 12 datasets is provided in Table 3. It can be observed that the proposed feature augmentation module and multi-task loss function achieve better performance among most datasets. Moreover, it improves the baseline in terms of the HM scores of 12 datasets by 23.51%, 32.47%, 4.28%, −1.23%, 0.02%, 0.42%, 1.85%, 3.66%, 8.74%, −1.11%, 0.4%, and 0.25%, respectively. These experimental results indicate that the early classification performance of the model is effectively improved through these two components.

#### 4.3.2. Ablation Experiments of Gradient Projection Technique

In this section, the ablation experiments are performed to illustrate the effectiveness of the gradient projection technique. Specifically, we use the TCN backbone (with our loss function) as the baseline, and then the baseline with (DETSCNet) and without gradient projection technique (abbreviated to DETSCNet (without GP)) are compared. The quantitative results of these experiments are provided in Table 4. As presented, the performance of early classification is improved by introducing the gradient projection technique. Moreover, it improves the DETSCNet (without GP) in terms of the HM scores of 12 datasets by 2.68%, 1.72%, 0.74%, 0.13%, 0.25%, 4.18%, 1.04%, 0.58%, 8.08%, 0.71%,0.87%, and 0.19%, respectively. The experiment results demonstrate the effectiveness of the gradient projection technique.

## 5. Discussion

In this section, the detailed analysis of the proposed method will be provided to illustrate the impact of different modules on ETSC.

### 5.1. Varied-Length Time Series Classification

**Figure 6:**
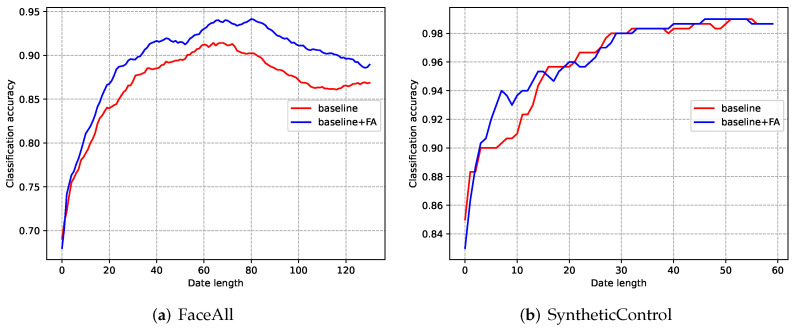
The curves classification accuracy of the model with and without the feature augmentation module for the FaceAll dataset and SyntheticControl dataset.

To illustrate the improvement brought by the feature augmentation modules and the multi-task loss function, we present the classification accuracy with regard to the data length in Figure 6. As shown, the model with the feature augmentation module (baseline + FA) performs obviously better than the model without this module (baseline). It can be found that the classification performance improves when the data length is relatively short. This ensures satisfied classification performance when the exiting subnet quits at an earlier time.

### 5.2. The Conflict between Varied-Length Time Series Classification Task and Early Exiting Task

**Figure 7:**
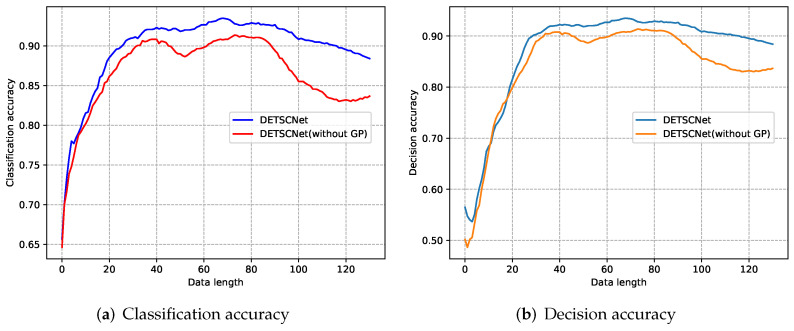
The curves of classification accuracy and decision accuracy with regard to data length for DETSCNet and DETSCNet (without GP) on the FaceAll dataset.

To illustrate the impact of the gradient projection technique for ETSC, the curves of the classification accuracy with regard to data length for DETSCNet and DETSCNet (without GP) are shown in Figure 7a. Moreover, the curves of decision accuracy with regard to data length are shown in Figure 7b. The decisions of the exiting subnet are considered correct, if the quitting decisions are consistent with the VTSC results (exiting with correct classification, and not exiting with false classification). It can be observed that, after performing the gradient projection, both the exiting and classification performance is increased. This may be because the gradient projection technique mitigates the conflict between the classification and early quitting.

**Figure 8:**
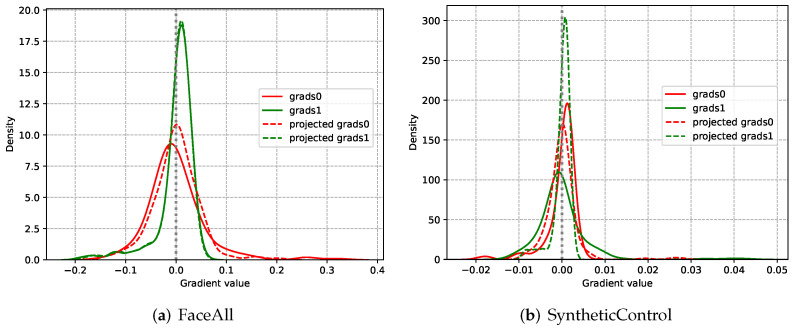
The gradient distribution of the parameters of the first convolutional layer. The grads0 and grads1 represent the raw gradient of the VTSC task and early exiting task, respectively. The dotted line represents the gradient distribution of these parameters after gradient projection. The peaks of the curves represent the gradient direction of most parameters.

Besides, we separately calculate the gradients of the first convolutional layer with regard to these two tasks. The distribution curves of the calculated gradients are presented in Figure 8. As shown in Figure 8a, before performing the gradient projection, the peak of the solid red curve with regard to the VTSC task is located at the left side of the 0 axis. Moreover, the peak of the solid green curve with regard to the early exiting task is located at the right side of the 0 axis. The peaks of the curves represent the gradient direction of most parameters. It can be concluded that most gradients of the classification task and early exiting task have opposite directions due to their conflicting objectives. After the gradient projection, the peaks corresponding to the two tasks (dotted lines) are both changed to the right side of the 0 axis. Therefore, the conflicting gradients are projected into a unified direction. Similar phenomena can also be observed in Figure 8b. These visualization results indicate that the proposed gradient projection technique effectively tackles the conflict between the classification and the early exiting.

## 6. Conclusions

In this paper, we decoupled the ETSC task into the VTSC task and the early exiting task and proposed an end-to-end framework (DETSCNet) composed of the classification and exiting subnets. First, a varied-length feature augmentation module and a specially designed loss function were proposed. In this way, the adaptive capability of the classification subnet to the length variation of flow data can be enhanced. Then, the gradient projection technique was applied to project the conflicting gradients into a unified direction, which handles the conflict between classification and early exiting. Our proposed DETSCNet achieved superior performance compared with the results of competitors. In future work, we will further explore how to enhance the data adaptation capability of deep models.

## Figures and Tables

**Table 1:** Configurations of the model.

Layer	Dilation Factor	Kernel Size	Number of Features
Temporal convolutional module1	1	3	64
Temporal convolutional module2	2	3	64
Temporal convolutional module3	4	3	64
Pooling layer	∖	∖	∖
Linear layer of VTSC subnet	∖	∖	∖
Linear layer exiting subnet	∖	∖	∖

**Table 2:** Quantitative HM (%) score results of the different methods on the 12 datasets.

Dataset	SR2-CF2	ECLN	ETMD	EPTS	DETSCNet
ChlorineCon	**71.13**	*69.59*	69.35	48.56	68.12
CricketX	*64.35*	56.57	26.02	52.04	**64.49**
FaceAll	80.01	16.84	7.65	*86.13*	**87.94**
MedicalImages	**81.33**	52.08	4.86	51.25	*80.27*
NonInvThorax2	*87.52*	80.31	0.15	73.94	**89.47**
StarLightCurves	*91.51*	29.57	81.17	80.28	**92.86**
SyntheticControl	*84.62*	22.83	29.59	91.29	**93.88**
TwoPatterns	17.00	17.42	42.84	*57.66*	**61.85**
UWaveZ	59.96	41.03	27.87	*61.24*	**62.99**
Wafer	*95.76*	86.01	94.00	94.87	**98.78**
HHAR	81.91	53.27	73.12	*94.40*	**96.63**
DSA	81.28	24.47	53.93	*95.78*	**99.29**

**Table 3:** Quantitative HM (%) score results of the ablation experiments for the designed feature augmentation module and multi-task loss function.

Datasets	Baseline	Baseline + FA
ChlorineCon	41.93	**65.44**
CricketX	30.3	**62.77**
FaceAll	82.92	**87.2**
MedicalImages	**81.37**	80.14
NonInvThorax2	89.2	**89.22**
StarLightCurves	88.26	**88.68**
SyntheticControl	90.99	**92.84**
TwoPatterns	57.61	**61.27**
UWaveZ	46.17	**54.91**
Wafer	**99.18**	98.07
HHAR	95.36	**95.76**
DSA	98.85	**99.1**

**Table 4:** Quantitative HM (%) score results of the ablation experiments for the gradient projection technique.

Datasets	DETSCNet (without GP)	DETSCNet
ChlorineCon	65.44	**68.12**
CricketX	62.77	**64.49**
FaceAll	87.2	**87.94**
MedicalImages	80.14	**80.27**
NonInvThorax2	89.22	**89.47**
StarLightCurves	88.68	**92.86**
SyntheticControl	92.84	**93.88**
TwoPatterns	61.27	**61.85**
UWaveZ	54.91	**62.99**
Wafer	98.07	**98.78**
HHAR	95.76	**96.63**
DSA	99.1	**99.29**

## Data Availability

Publicly available datasets were analyzed in this study. This data can be downloaded from the following links: http://www.timeseriesclassification.com/ (UCR datasets, accessed on 15 August 2021), http://archive.ics.uci.edu/ml/datasets/heterogeneity+activity+recognition (HHAR dataset, accessed on 24 August 2021) and https://archive.ics.uci.edu/ml/datasets/daily+and+sports+activities (DSA dataset, accessed on 24 August 2021).
